# MeSRT1 Interacts with MeNF-YC15 and Negatively Mediates Resistance to Cassava Bacterial Blight via Regulation of Gibberellin Metabolism in *Manihot esculenta* Crantz

**DOI:** 10.3390/plants15192898

**Published:** 2026-09-22

**Authors:** Yujing Bai, Lan Yang, Yongmin Liu, Wenlei Yan, Hongqiu Zeng

**Affiliations:** 1 Sanya Nanfan Research Institute of Hainan University, Key Laboratory of Biotechnology of Salt Tolerant Crops of Hainan Province, School of Tropical Agriculture and Forestry, Hainan University, Haikou 570228, China; 2 Guizhou Institute of Horticulture, Guizhou Academy of Agricultural Sciences, Guizhou Horticultural Engineering Technology Research Center, Guiyang 550006, China

**Keywords:** cassava, sirtuin, disease resistance, gibberellin

## Abstract

Cassava (*Manihot esculenta* Crantz) has significant economic value as a vital source of food, feed, and bio-energy. However, the production of cassava is severely restricted by cassava bacterial blight (CBB). Sirtuins (SRTs) are conserved histone deacetylases that play essential roles in the plant energy metabolism and stress response. However, the family of SRTs has not been identified in cassava. In this study, we isolated and identified three MeSRTs in response to CBB infection and found that they were negative regulators of cassava resistance using a transient gene-silencing assay. Further studies found that MeSRT1 physically interacted with the transcription factor Nuclear Factor YC15 (MeNF-YC15). Transient silencing and overexpression assays suggested that MeSRT1 negatively regulated the transcription of *gibberellin 2-oxidases 1*/*2* (*MeGA2ox1/2*), which promoted the accumulation of gibberellic acid 1 (GA1). However, MeNF-YC15 promoted the transcription of *MeGA2ox1/2* and further increased the metabolism of GA1. Taken together, MeSRT1 and MeNF-YC15 antagonistically regulated the GA1 content and resulted in an antagonistic effect on cassava disease resistance. This study identified a MeSRT1-MeNF-YC15 molecular module in CBB resistance responses by regulating the GA1 content in cassava.

## 1. Introduction

Cassava (*Manihot esculenta* Crantz) is an essential tuberous root crop that is rich in starch [1]. It has been widely cultivated in tropical and subtropical regions and regarded as a staple food and income source for more than 800 million residents [2]. In addition, the products of cassava, such as starch and bio-alcohol, are important for the green economy and biological energy industry [3]. However, cassava-growing regions are often threatened by cassava bacterial blight (CBB), caused by *Xanthomonas axonopodis* pv. *manihotis* (*Xam*), which is also referred to as *Xanthomonas phaseoli* pv. *manihotis* (*Xpm*) [4,5]. CBB is a devastating bacterial disease threatening cassava cultivation and leading to a 30–100% loss in cassava yield [5]. After CBB infection, leaves suffer from water-soaked lesions, chlorosis, defoliation, and stem browning, which can even result in plant death [6]. Cultivating high disease-resistance cassava varieties is the most effective method for CBB control, compared with field control measurements [7]. Thus, some disease-resistant genes have been isolated and identified in recent years. For example, Díaz et al. [8] identified and cloned the *Resistance to Xam 1* (*RXam1*) gene, encoding a receptor-like kinase (RLK) through BAC library screening. They demonstrated that the overexpression of *RXam1* could significantly enhance the disease resistance to *XamCIO136* in susceptible cassava plants [8]. Since then, *RXam2* has also been identified, which inhibits the growth of various *Xpm* strains and alleviates CBB symptoms [9]. In recent years, some cassava susceptibility (S) genes have also been discovered. For example, the methylation of the promoter of the S gene *Sugars Will Eventually be Exported Transporter 10a* (*MeSWEET10a*) could further inhibit the transcription of *MeSWEET10a*, which resulted in mild CBB symptoms [10]. Similarly, CRISPR/Cas9 generated Transcription Activator-like 10 (TAL10) binding site-edited cassava plants blocked the induction of *MeSWEET10a*, which reduced the susceptibility of cassava to CBB [11]. Despite these advances, the existing gene resources and the genetic mechanism of CBB resistance are relatively limited. More studies are required to accelerate the precise breeding of cassava CBB-resistant varieties.

Sirtuins are a conserved family of nicotinamide adenine dinucleotide NAD^+^-dependent histone deacetylases [12]. Sirtuins can catalyze the removal of acetyl residues of numerous target proteins (histone and non-histone), and function as important regulators of cellular physiology [13]. Sirtuins were first isolated in yeast (*Saccharomyces cerevisiae*), and seven sirtuins were subsequently isolated in mammals [14]. The mammalian sirtuins are involved in histone deacetylation and transcriptional regulation, thereby regulating various life processes such as lifespan extension, DNA repair, metabolic process and stress response, and function as key metabolic receptors and epigenetic regulators [15]. The research on plant sirtuins (SRTs) was later compared with that on animal sirtuins [16]. However, it is interesting that the homologues of SRTs in plants are much fewer than those in animals. For examples, only two SRT homologues were identified in several common plants, such as *Arabidopsis thaliana*, rice (*Oryza sativa* L.), and tomato (*Solanum lycopersicum* L.) [17,18,19]. Functional studies show that SRTs play an important role in plant energy metabolism through transcriptional regulation. For example, the overexpression of *FaSRT2-1* could promote the accumulation of organic metabolites, including soluble sugars and anthocyanins [20]. AtSRT1 could interact with cMyc-Binding Protein 1 (AtMBP-1) and further inhibit the transcription of glycolytic genes, thereby negatively regulating glycolysis in *Arabidopsis* [21]. Meanwhile, OsSRT1 was also found to inhibit glycolysis by repressing the moonlighting function of Glyceraldehyde-3-phosphatedehydrogenase (GAPDH), which serves as an important transcriptional activator of glycolysis [22]. SRTs are also involved in plant stress defense through transcription regulation. For example, AtSRT2 deacetylated Vesicle-associated membrane protein 714 (VAMP714) and inhibited its transcriptional activity, which further improved its defense against excessive H_2_O_2_ accumulation and salt tolerance during seed germination [23]. AtSRT1 was also involved in glycolysis in *Arabidopsis*, thereby further regulating the plant stress defense [21]. Taken together, SRTs are closely related to transcriptional regulation and play important roles in plant energy metabolism and stress responses.

An increasing number of studies have focused on the relationship between SRTs and plant immune responses in recent years. However, the functions and mechanisms of SRTs in plant disease responses remain unclear. Some studies suggest that SRTs were closely related to the transcription of many stress-related genes. For example, many transcription factors, antioxidant genes, and stress-related genes were induced in *FaSRT1-2* overexpressed strawberry, which decreased the resistance to *Botrytis cinerea* infection [24]. OsSRT2 could reduce the acetylation levels of H4K5ac and H4K8ac, thereby inhibiting the transcription of many defense genes, including *Os11g02540*, *Os08g28890*, *Os11g10710*, and *Os04g14690* [25]. Similarly, TaSRT1 could interact with and deacetylate the heat shock protein TaHSP18.6, thereby inhibiting the expression of *TaIAA1*, which enhanced the wheat resistance to crown rot [26]. Although the role of SRTs in plant disease resistance has been preliminarily explored in some plants, the understanding of SRTs in the plant immune regulatory network is still limited, and their biological functions and molecular pathways in cassava remain to be further clarified.

Nuclear factor Y (NF-Y) is a conserved transcript factor in plants, and plays important roles in plant immune responses [27]. Previous studies suggested that they were involved in phytohormone responses in plants. For example, OsNF-YAs could suppress the transcription of many jasmonic acid (JA)-related genes, enhancing the susceptibility to the Rice stripe virus and Southern rice black-streaked dwarf virus [28]. Jiang et al. [29] identified 25 NF-Ys in watermelon (*Citrullus lanatus* L.) and found that most of them responded significantly to salicylic acid (SA), methyl jasmonate, abscisic acid (ABA), and 1-aminocyclopropane-1-carboxylate (the precursor of ethylene). In potato (*Solanum tuberosum* L.), StNF-YA8 could increase the accumulation of gibberellin (GA) and ABA by activating the transcription of their biosynthesis genes [30]. Among these phytohormones, JA, SA, and ethylene have long been considered as key regulators in plant immunity [31]. However, recent studies have indicated that GA also plays essential roles in plant immunity [32,33]. NF-Y was closely related to many phytohormones; however, whether NF-Y regulates phytohormones, especially GA, in cassava and the underlying mechanisms remains to be further studied.

In this study, we analyzed the phylogenetic tree of the cassava *MeSRT* gene family and identified *MeSRT1* as a negative regulator of CBB resistance. A molecular mechanism analysis revealed that MeSRT1 can interact with MeNF-YC15 and participate in the regulation of CBB resistance by mediating the expression of GA-metabolism-related genes. This study not only expanded the functional understanding of plant SRT family proteins, but also provided new potential genetic resources for CBB resistance breeding, providing new insights into the sustainable control of cassava CBB diseases.

## 2. Results

### 2.1. MeSRTs Negatively Regulate CBB Resistance in Cassava

To identify SRTs in cassava, the protein sequences of SRTs from *Arabidopsis*, rice, and cassava were obtained from databases and then subjected to BLAST searches against the Phytozome database. Three MeSRTs with conserved domains (IPR003000:Sirtuin family; IPR026590: Sirtuin family, catalytic core domain; IPR029035: DHS-like NAD/FAD-binding domain) were identified. Then, the evolutionary tree was constructed and SRTs in cassava were identified and named MeSRT1, MeSRT2, and MeSRT3 according to their genetic relationship (Figure 1A). MeSRT1 clusters with AtSRT1 in the same branch, while MeSRT2 and MeSRT3 are more similar to AtSRT2 and OsSRT2 (Figure 1A), suggesting a close genetic relationship among SRTs from *Arabidopsis*, rice, and cassava. To explore the function of *MeSRTs* in response to CBB, the expression patterns of *MeSRTs* after *Xpm* infection were analyzed. *MeSRT1* and *MeSRT2* were gradually upregulated after *Xpm* infection, indicating their potential roles in the cassava response to CBB infection (Figure 1B).

To further identify the function of MeSRTs in cassava disease resistance, three *MeSRTs* were individually silenced and inoculated with *Xpm*, respectively (Figure 2A). At 4 days post-infection (dpi), *MeSRT1-*, *MeSRT2-*, or *MeSRT3-*silenced cassava plants showed milder disease phenotypes, less bacterial numbers, and less lesion areas than CK (Figure 2B–D), indicating that MeSRTs negatively regulate the resistance to CBB in cassava.

### 2.2. MeSRT1 Interacts with MeNF-YC15

Although MeSRTs are involved in cassava disease resistance, the possible mechanisms by which they regulate cassava disease resistance remain unclear. Thus, considering the improved phenotype of MeSRT1 in regulating cassava disease resistance, the CDS of *MeSRT1* was cloned into the pGBKT7 vector and a yeast two-hybrid library screening assay was performed to identify potential proteins interacting with MeSRT1. In this way, MeNF-YC15 was screened. Considering the important roles of NF-Ys in plant disease resistance [27], MeSRT1 may regulate cassava disease resistance through protein interaction. To further identify this result, the CDS of *MeNF-YC15* was cloned into the pGADT7 vector and co-transformed into yeast with *MeSRT1-*pGBKT7. The transformed yeast was then inoculated onto selective medium. Subsequently, the *MeSRT1-*pGBKT7 and *MeNF-YC15*-pGADT7 co-transformed yeast was grown on SD/−Leu−Trp−His−Ade medium, whereas the negative control did not grow (Figure 3A), suggesting that MeSRT1 interacts with MeNF-YC15. Then, a pull-down assay was performed to further verify the protein interaction. MeSRT1-Flag and MeNF-YC15-Myc protein were induced by *E. coli* BL21; then, the proteins were mixed and immunoprecipitated with anti-Myc antibody. After chemical development processes, the protein band of MeSRT1-Flag in the IP group was observed, whereas no band was observed in the negative control, indicating a protein interaction between MeSRT1 and MeNF-YC15 in vitro (Figure 3B). Meanwhile, MeSRT1-cYFP and MeNF-YC15-nYFP were overexpressed in tobacco, and the MeSRT1-cYFP + MeNF-YC15-nYFP group showed yellow fluorescence in the nucleus, suggesting that MeSRT1 interacts with MeNF-YC15 in vivo (Figure 3C). In conclusion, MeSRT1 and MeNF-YC15 interact with each other both in vitro and in vivo.

### 2.3. MeSRT1 and MeNF-YC15 Antagonistically Regulates Disease Resistance in Cassava

Although the interaction between MeSRT1 and MeNF-YC15 was identified, how this interaction functions in cassava defense remained unclear. To verify this hypothesis, *MeSRT1* or *MeNF-YC15* was silenced and inoculated with *Xpm*, respectively (Figure 4A). Then, *MeNF-YC15*-silenced cassava plants showed more severe disease phenotypes than CK, whereas the disease phenotypes of *MeSRT1-*silenced cassava plants were milder than those of CK (Figure 4B). Meanwhile, the *MeSRT1-* and *MeNF-YC15-*co-silenced cassava plants showed phenotypes similar to those of CK (Figure 4B). These results were further supported by the quantification of bacterial number and lesion area (Figure 4C,D). Taken together, MeSRT1 and MeNF-YC15 might act as a switch and antagonistically regulate disease resistance in cassava.

### 2.4. MeSRT1 and MeNF-YC15 Commonly Regulate the Transcription of GA-Related Genes

Previous studies suggested that SRTs and NF-Ys play important roles in regulating the transcription of downstream genes [34,35]. Therefore, an RNA-seq analysis was performed to identify their downstream genes and investigate the mechanisms underlying cassava disease defense. About 3581 differentially expressed genes (DEGs) (screened by |log_2_Fold change| ≥ 1 and adjusted *p* value < 0.05) were identified in *MeSRT1-*silenced cassava plants and 544 DEGs were identified in *MeNF-YC15-*silenced cassava plants (Figure 5A). In order to identify downstream genes commonly regulated by MeSRT1 and MeNF-YC15, the DEGs in *MeSRT1-*silenced cassava or in *MeNF-YC15-*silenced cassava were compared, and 194 DEGs were found to exist in both groups (Figure 5A). To ensure the reliability of this overlap, Fisher’s exact test and hypergeometric test were performed and the *p* values from both methods were less than 0.05, suggesting that this overlap was statistically significant. Therefore, these 194 DEGs were regarded as potential downstream genes commonly regulated by MeSRT1 and MeNF-YC15 (Figure 5A). Among these DEGs, 13 DEGs were closely related to phytohormone responses (Figure 5B). This enrichment was also examined by Fisher’s exact test and the hypergeometric test, and both tests were significant (*p* < 0.05), suggesting that MeSRT1 and MeNF-YC15 might commonly regulate the gibberellic acid, ethylene, and auxin-related pathway. Among these genes, only Manes.15G002900 (*MeGA2ox2*) and Manes.03G208300 (*MeGA2ox1*) have an exact function in GA and Manes.03G166300 has an exact function in ethylene. Moreover, the |log_2_(Fold change)| in *MeGA2ox1* in *MeSRT1-*silenced cassava was the largest among them. Gibberellin 2-oxidases catalyze the conversion of bioactive GA1 or GA4 to inactive GAs [36], and bioactive GA has been described as key regulator of plant immunity in recent years [37]. A previous study suggest that bioactive GA reduces cassava disease resistance [37]; it is possible that MeSRT1 and MeNF-YC15 regulate cassava disease resistance through *MeGA2ox1-* and *MeGA2ox2-*mediated GA1 and GA4 metabolism. Thus, *MeGA2ox1* and *MeGA2ox2* were chosen for further study.

### 2.5. MeSRT1 and MeNF-YC15 Regulate GA1 Metabolism Instead of GA4 Metabolism

To verify this speculation, the transcript levels of *MeGA2ox1* and *MeGA2ox2* were first verified in *MeSRT1-* or *MeNF-YC15-*silenced cassava plants. The results showed that the transcript levels of *MeGA2ox1* and *MeGA2ox2* were increased in *MeSRT1*-silenced cassava, but decreased in *MeNF-YC15*-silenced cassava (Figure 6A,B), consistent with the RNA-seq result. Moreover, the GA1 content was reduced in *MeSRT1-*silenced cassava plants, but increased in *MeNF-YC15*-silenced cassava (Figure 6C). However, the GA4 content did not change when *MeSRT1* or *MeNF-YC15* was silenced (Figure 6D). Meanwhile, the transcript levels of *MeGA2ox1* and *MeGA2ox2* were detected, and they were higher than those in CK when *MeNF-YC15* was overexpressed but decreased when *MeSRT1* was overexpressed (Figure 6E,F). The GA1 content was reduced when *MeNF-YC15* was overexpressed, and increased when *MeSRT1* was overexpressed (Figure 6G). However, the GA4 content did not change in these samples (Figure 6H). Taken together, these results suggest that MeSRT1 and MeNF-YC15 commonly regulate the GA1 content by modulating the expression of *MeGA2ox1* and *MeGA2ox2*.

### 2.6. MeSRT1 and MeNF-YC15 Regulate Disease Resistance Through GA1

To verify whether MeSRT1 and MeNF-YC15 regulate cassava resistance through GA1 metabolism, an *Agrobacterial-*mediated *MeNF-YC15* transient overexpressed assay was performed in *MeSRT1-*silenced cassava to generate *MeSRT1-TRV* and *MeNF-YC15-OE* samples (Figure 7A). These samples showed a significantly lower GA1 content than CK (Figure S1). Then, these samples were treated with H_2_O or GA1, and inoculated with *Xpm*. The disease phenotypes of *MeSRT1-TRV* and *MeNF-YC15-OE* samples were milder than those of CK; however, the disease phenotypes became more severe after GA1 treatment (Figure 7B–D). These results indicated that GA1 treatment could increase the susceptible of cassava to diseases. Meanwhile, the disease phenotypes of GA1-treated *MeSRT1-TRV* and *MeNF-YC15-OE* samples were slightly weaker than those of GA1-treated CK (Figure 7B–D). These results suggest that MeSRT1 and MeNF-YC15 regulate cassava disease resistance by modulating GA1 metabolisms, to some extent.

## 3. Discussion

Sirtuins are essential enzymes involved in multiple essential processes in plants, such as gene transcription, the regulation of proteins activity, and metabolism [38,39]. However, the function and mechanisms of sirtuins in cassava disease resistance were unclear. In this study, the cassava sirtuin gene family was identified. Then, the mechanism by which MeSRT1 interacts with MeNF-YC15 and further regulates GA1 metabolism was elucidated, revealing the molecular mechanism of MeSRT1 in cassava disease resistance.

Some studies suggested that sirtuins regulate plant resistance to external stresses through phytohormones. In *Brassica napus*, *BnaSRTs* responded significantly to ABA, JA, and IAA, which play important roles in plant responses to various abiotic or biotic stresses [40]. SRT1 and SRT2 could interact with EIN2 Nuclear Associated Protein 1 (ENAP1) and further regulate the transcription of many ethylene signaling genes in *Arabidopsis* [41]. AtSRT1 could suppress the transcription of several ABA signaling genes, including *Low expression of osmotically responsive genes 2* (*LOS2*), *cMyc-binding protein 1* (*MBP1*), and *Zinc finger protein (ZAT10*), and further regulate the tolerance of *Arabidopsis* to various stresses [21]. Sirtuins not only regulate phytohormone signal transduction but may also be involved in the regulation of phytohormone contents. For examples, Wang et al. [24] found that the ABA content decreased and GA content increased in *FaSRT1-2*-overexpressing strawberry (*Fragaria* × *ananssa* Duch.), which was susceptible to *Botrytis cinerea* infection. Similar to these results, we found that *MeSRT1*-silenced cassava had a lower GA1 content than CK, and were more resistant than CK. Thus, SRTs might commonly promote GA accumulation in different species, which contributes to the susceptibility to disease infection. Taken together, sirtuins are closely associated with phytohormones, and are involved in phytohormone signal transduction, biosynthesis, and metabolism.

In early studies, GA was considered as a key phytohormone in regulating plant development [42]. However, the important role of GA in plant disease resistance has also been recognized in recent years, and GA has been served as a central regulator coordinating plant growth and disease resistance [32,43]. For example, Qin et al. [44] found that *Gibberellin 20-oxidase 3* (*OsGA20ox3*)-overexpressing rice plants showed higher levels of bioactive GAs (GA1 and GA4), and were more susceptible to rice blast. The GA biosynthetic enzyme GA20ox1 could interact with Brassinazole-Resistant 6 (BZR6), coordinating brassinosteroids (BR)-mediated disease resistance and GA homeostasis [33]. The possible mechanism by which GAs influence disease resistance might be due to the DELLA proteins, which could be degraded under high GA conditions [37]. In fact, DELLA proteins could inhibit GA signaling, thereby resulting in growth inhibition. Meanwhile, DELLAs were reported to interact with many proteins involved in SA and JA signaling, which play important roles in plant immunity [43]. In this study, we found that MeSRT1 and MeNF-YC15 could promote the metabolism of GA1. Similar to these studies, the low level of GA1 resulted in enhanced disease resistance in cassava. These studies suggested that the homeostasis of bioactive GAs was essential for plant development and disease response. However, bioactive GAs might have different functions in different species in response to different disease [45,46]. Considering the diverse types of bioactive GAs and inactive GAs, the situations might be relatively complex [47]. Nevertheless, the important roles of GAs in coordinating plant development and disease response are well-established, and worthy of further studies.

Sirtuins have extensive regulatory effects on gene expression, including phytohormone biosynthesis genes [38]. Thus, sirtuins are involved in phytohormone biosynthesis and further mediate plant development and stress responses [24,26,48]. In our study, functional experiments carried out by transient silencing and overexpression suggest that sirtuins are also involved in the metabolism process of GA1, which is regulated by the interaction between MeSRT1 and MeNF-YC15. This protein interaction was identified in yeast, tobacco, and in vitro. This interaction antagonistically regulates the transcription of *MeGA2ox1/2*, thereby influencing cassava disease resistance through GA1 metabolism. This study proposes a novel MeSRT1-MeNF-YC15-*MeGA2ox1/2-*GA1 module for cassava disease resistance improvement (Figure 8).

## 4. Materials and Methods

### 4.1. Plant Materials and Growth Conditions

The cassava variety ‘SC124’ was obtained from Danzhou planting bases (Danzhou city, Hainan province, China) and grown in Yazhou city (Yazhou city, Hainan province, China). ‘SC124’ is a neutral variety to CBB. The stems of cassava were cut into segments containing about 2–4 buds, and then planted in standard pots filled with nutrient soil and vermiculite in equal proportions. The cassava plants were then grown in a greenhouse (16 h/8 h of light/night, 70–80% humidity, 26 °C) [49]. About 4–5-week old cassava plants were used for gene silencing or overexpressing.

### 4.2. Phylogenetic Tree Construction

The amino acid sequences of sirtuins in cassava, *Arabidopsis*, and rice were obtained from Phytozome (v14) database “https://phytozome-next.jgi.doe.gov (accessed on 16 May 2023), TAIR database “https://www.arabidopsis.org (accessed on 16 May 2023), and China rice data center “https://www.ricedata.cn (accessed on 16 May 2023), respectively. The protein sequences were aligned by ClustalW method. A phylogenetic tree was constructed using the neighbor-joining (NJ) method by MEGA (v6.05) software with 1000 bootstrap replicates [50]. The information of MeSRTs is listed in Table S1.

### 4.3. Expression Analysis

*Xpm* (NCBI Taxonomy ID 43353) strain (*Xam* Hainan) used in this study was obtained from Hainan Key Laboratory for Sustainable Utilization of Tropical Bioresources [51]. For *Xpm* inoculation, the single colony of *Xpm* was cultured at 28 °C until OD_600_ = 0.6. The medium was then discarded and the bacteria were resuspended with 10 mM MgCl_2_ until OD_600_ = 0.1. The bacterial solution was inoculated into cassava leaves by 1 mL needle with 50 μL solution. The leaf samples were harvested at 0 h post infiltration (hpi), 3 hpi, 6 hpi, and 24 hpi. The 0 hpi samples were harvested immediately after inoculation.

Total RNA was extracted from cassava leaves using the RNAprep Pure Plant Kit (DP441, Tiangen, Beijing, China) according to the manufacturer’s instructions. Then, cDNA was generated using a reverse-transcription kit (AG11705, Accurate Biology, Changsha, China). The RT-qPCR analysis was performed using SYBR Green (Q711-02, Vazyme, Nanjing, China) and Bio-Rad CFX Connect system (CFX96, Bio-Rad, Hercules, CA, USA). The RT-qPCR programs were set as follows: (1) 95 °C 2 min; and (2) 95 °C 15s, 50–55 °C 15s, 72 °C 15s, 40 cycles. Relative transcript levels were calculated using the 2^−ΔΔCT^ method. In this method, CT was obtained from instrument, ΔCT = CT(target gene) − CT(reference gene), and ΔΔCT = ΔCT(experimental group) − ΔCT(control group). *EF1α* (Manes.15G054800) was used as the reference gene, which has been described as a stable reference gene during *Xpm* infection [52]. The primers used in RT-qPCR are listed in Table S2.

### 4.4. The Calculation of Bacterial Lesion and Bacterial Number

The *Xpm* inoculation method was performed as described in Section 4.3. For 0-day-post-*Xpm-*inoculation (0 dpi) samples, the leaves were harvested immediately after *Xpm* inoculation. Then, 4 days post *Xpm* inoculation (4 dpi), the inoculated leaves were photographed and the lesion area was calculated by ImageJ (v1.50i) software according to a previous study [53]. Then, leaf discs with 1 cm diameter from inoculated cassava leaf were collected and surface-sterilized with 75% ethanol. The collected leaves were then homogenized in sterile water and gradient-diluted onto LB agar plates. The plates were cultured at 28 °C for 24 h, and then the bacterial colonies were counted [54].

### 4.5. Gene Silencing and Overexpressing Assay

For gene silencing, the partial sequences of target genes were individually cloned into pTRV2 vector. The PCR programs used for cloning were set as follow: (1) 95 °C 3 min; and (2) 95 °C 30s, 50–55 °C 30s, 72 °C 1 kb/min, 29 cycles. The recombinant vectors were transformed into *Agrobacterium tumefaciens* GV3101 and the density was adjusted to OD_600_ = 0.6 with 10 mM 2-morpholinoethanesulfonic acid, 10 mM MgCl_2_, and 200 µM acetosyringone. Then, the bacterial suspension was inoculated into cassava leaves [37]. For the silencing assay, *Agrobacterium* carrying pTRV1 vector and *Agrobacterium* carrying recombinant pTRV2 vector were inoculated into cassava together. For the co-silencing assay, two different *Agrobacterium* carrying different recombinant pTRV2 vector were co-inoculated into cassava with pTRV1-transformed *Agrobacterium.* For the CK sample, pTRV1-transformed *Agrobacterium* was co-infiltrated with empty pTRV2-transformed *Agrobacterium.* At 14 days after *Agrobacterium* inoculation, the leaves were harvested and the transcript levels were analyzed as described in Section 4.3. The primer sequences used for RT-qPCR and vector construction are listed in Table S2 and Table S3, respectively.

For gene overexpression, the full length CDS of target genes were cloned into pEGAD vector. The PCR programs used for cloning were set as follows: (1) 95 °C 3 min; and (2) 95 °C 30s, 50–55 °C 30s, 72 °C 1 kb/min, 29 cycles. Inoculation method was referred to gene silencing. For CK sample, empty pEGAD-transformed *Agrobacterium* was infiltrated into cassava. Two days after *Agrobacterium* inoculation, leaves were harvested and transcript levels were analyzed as described in Section 4.3. Primer sequences used for RT-qPCR and vector construction are listed in Table S2 and Table S3, respectively.

### 4.6. Yeast Two-Hybrid (Y2H)

For the Y2H-screening assay, the CDS of *MeSRT1* was cloned into pGBKT7 vector, and co-transformed into yeast AH109 with library vectors. The library vectors were constructed by connecting cDNA fragments to pGADT7 vector. The transformation was based on PEG/LiAc method as described previously [55]. Briefly, the yeast was cultured until OD_600_ = 0.4–0.6, then centrifuged and resuspended in 1×TE/LiAc. The recombinant vectors were then added to resuspended yeast cells with PEG/LiAc. The mixture was incubated at 30 °C for 30 min and then at 42 °C for 15 min. The mixture was centrifuged and resuspended in YPDA medium, then shaken at 30 °C for 1 h. Finally, the transformed yeast cells were grown in the selective SD/−Leu−Trp−His−Ade medium at 30 °C. One week later, yeast cells were grown in selective medium. The colony was then cultured and the vectors were extracted. After sequencing and blasting, the potential interacting protein was identified as MeNF-YC15.

For the Y2H-verified assay, the CDS of *MeNF-YC15* was cloned into pGADT7 vector. Then, *MeSRT1-*pGBKT7 and *MeNF-YC15-*pGADT7 recombinant vectors were co-transformed into yeast AH109 using the PEG/LiAc method. The transformed yeasts were grown on selective SD/−Leu−Trp and SD/−Leu−Trp−His−Ade medium at 30 °C to verify protein interaction.

### 4.7. Pull-Down Assay

The CDS of *MeSRT1-Flag*, *MeNF-YC15-Myc*, and *GFP-Flag* were cloned into the pET32a vector, respectively. The sequences of Flag and Myc were based on previous studies [56,57]. All these constructs were transformed into *E. coli* BL21 (DE3) (ZC121-2, Weidi, Shanghai, China), respectively. Subsequently, the transformed *E. coli* cells were incubated with 1 mM isopropyl β-D-thiogalactoside (IPTG) at 28 °C to induce MeSRT1-Flag, MeNF-YC15-Myc, and GFP-Flag proteins. Then, the recombinant proteins were mixed, and MeNF-YC15-Myc + GFP-Flag group was used as control group. The samples were then incubated with Protein A/G MagBeads (L00277, Genscript, Nanjing, China) and anti-Myc antibodies (AM926, Beyotime, Shanghai, China) at 4 °C overnight. The beads were washed 5 times, and boiled in SDS-PAGE loading buffer, and then subjected to Western blot analysis. Anti-Myc (AM926, Beyotime, Shanghai, China) and anti-Flag (AF519, Beyotime, China) antibodies were used for detection.

### 4.8. BiFC Assay

The CDS (without stop codons) of *MeSRT1* and *MeNF-YC15* were cloned into cYFP vector and nYFP vector, respectively. In *MeSRT1-cYFP* vector, the sequences of *MeSRT1* were linked to part of the yellow fluorescent protein sequence. In *MeNF-YC15-nYFP* vector, the sequences of *MeNF-YC15* were linked to another part of yellow fluorescent protein sequence. These fused *MeSRT1-cYFP* or *MeNF-YC15-nYFP* were expressed under the control of 35S promoter through an *Agrobacterial-*mediated transient overexpression assay in tobacco leaves. Then, the leaves were observed and photographed using a confocal laser-scanning microscope (TCS SP8, Leica, Heidelberg, Germany).

### 4.9. RNA Sequencing and Analysis

Leaves from non-inoculated gene silencing cassava plants were collected in triplicate and used for total RNA extraction using CTAB (Bio Basic, Toronto, ON, Canada). Then, cDNA was then generated for library construction using Optimal Dual-mode mRNA Library Prep Kit (BGI-Shenzhen, Shenzhen, China). The libraries were sequenced on the BGISEQ-500 platform (BGI-Shenzhen, China), generating clean reads. The reads were then used for genome alignment against *Manihot esculenta* V7.1 using HISAT (v2.2.1), and gene annotation using Bowtie2 (v2.4.5), and differentially expressed genes analysis using DEseq2 (v1.40.2), as described previously [58,59,60]. Fisher’s exact test and the hypergeometric test were performed using R studio with “fisher.test” and “phyper” function, respectively.

### 4.10. Measurement of GA Contents and the Treatment of GA1

GA1 and GA4 contents were measured using Enzyme-Linked Immunosorbent Assay Kits (MM-1367Z1 and MM-1376Z1, Meimian, Yancheng, China). The measuring range of GA1 is 7.5–120 pmol/L. The measuring range of GA4 is 5–80 pmol/L. Variable coefficient was lower than 15%. The measurement procedures were performed as described previously [61]. Briefly, fresh cassava leaves were sampled and ground in liquid nitrogen, and then ground in cold PBS (0.01M, pH = 7.4) solution using 0.5 g of samples per 5 mL PBS solution. The homogenate was incubated at 4 °C for 3 h with shaking. Subsequently, the supernatant was collected after centrifugation at 5000 g for 5 min. GA1 and GA4 contents were then measured according to the manufacturer’s instructions.

For GA1 treatment, 100 μM GA1 was mixed with 0.01% Silwet L-77 surfactant, and then sprayed onto cassava leaves until the leaves were fully moistened. The treatment was performed every 12 h for a total of four times. At 8 h after final treatment, the leaves were inoculated.

### 4.11. Statistical Analysis

Statistical significance was evaluated using Duncan’s multiple-range test (*p*  <  0.05) following one-way ANOVA (SPSS, v21). The experiments were performed with at least three independent replicates. All data were based on at least three biological replicates, and different letters indicate the significance differences among groups.

## 5. Accession Number

Gene numbers are listed as follows: *AtSRT1* (*AT5G55760*), *AtSRT2* (*AT5G09230*), *OsSRT1* (*Os04g0271000*), *OsSRT2* (*Os12g0179800*), *MeSRT1* (*Manes.03G14140*0), *MeNF-YC15* (*Manes.01G245200*), *MeGA2ox1* (*Manes.03G208300*), and *MeGA2ox2* (*Manes.15G002900*).

## Figures and Tables

**Figure 1:**
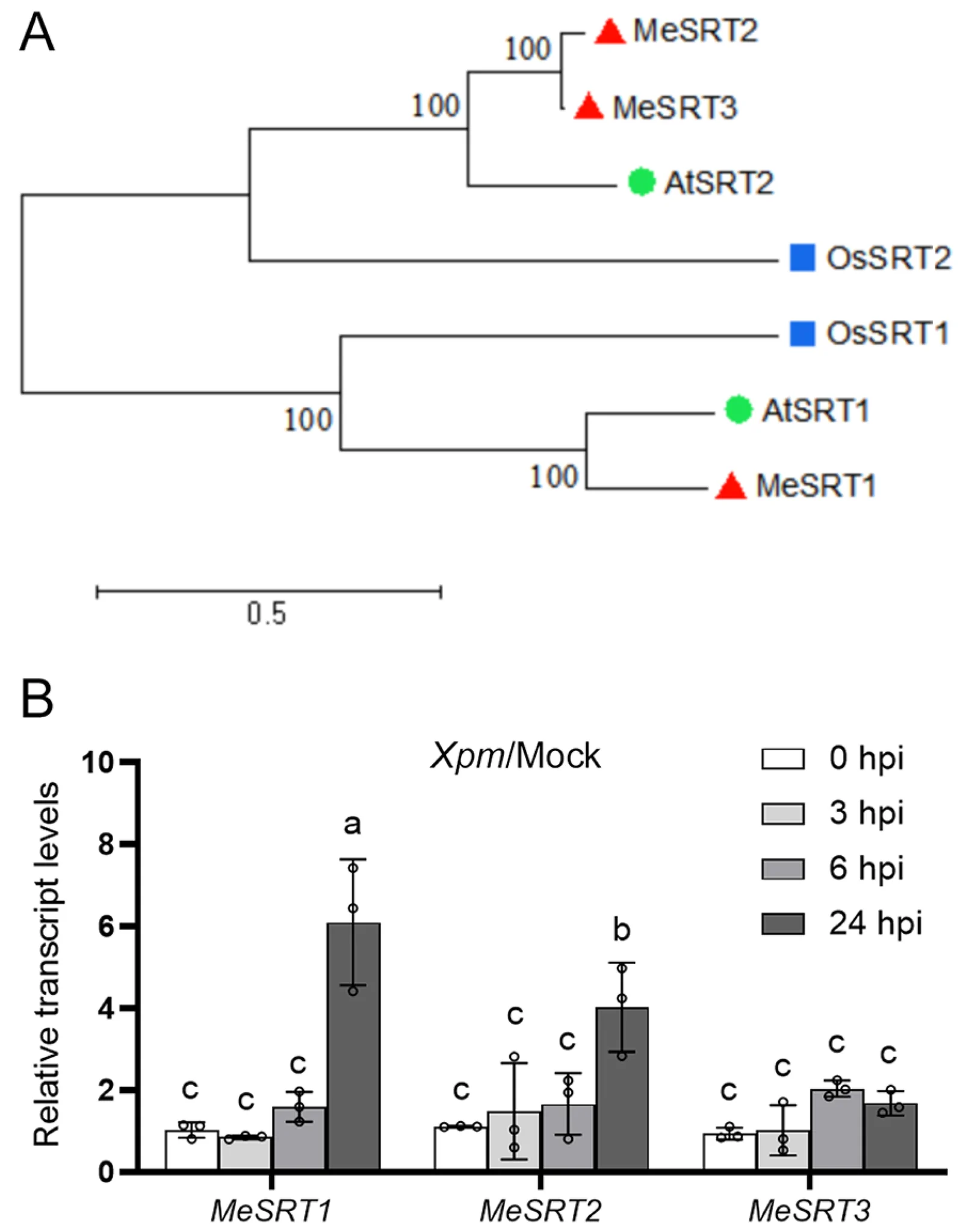
Genetic relationships of *MeSRTs* and their responses to *Xpm* infection. (**A**) Evolutionary tree of SRTs. The protein sequences of SRTs from *Arabidopsis*, rice (*Oryza sativa* L.), and cassava (*Manihot esculenta* Crantz) were obtained from databases and analyzed by MEGA (v6.05). The numbers represent bootstrap result, indicating phylogenetic relationship. The scale bar represents degree of actual evolutionary divergence. Green circles, blue squares, and red triangles indicate the proteins from *Arabidopsis*, rice, and cassava, respectively. (**B**) The relative transcript levels of *MeSRTs* in response to *Xpm* infection. Results are presented as mean ± SD (*n* = 3). Mock refers to cassava inoculated with MgCl_2_. Significant differences were calculated by Duncan’s range test using SPSS (v21). Different letters represent significant differences (*p* < 0.05).

**Figure 2:**
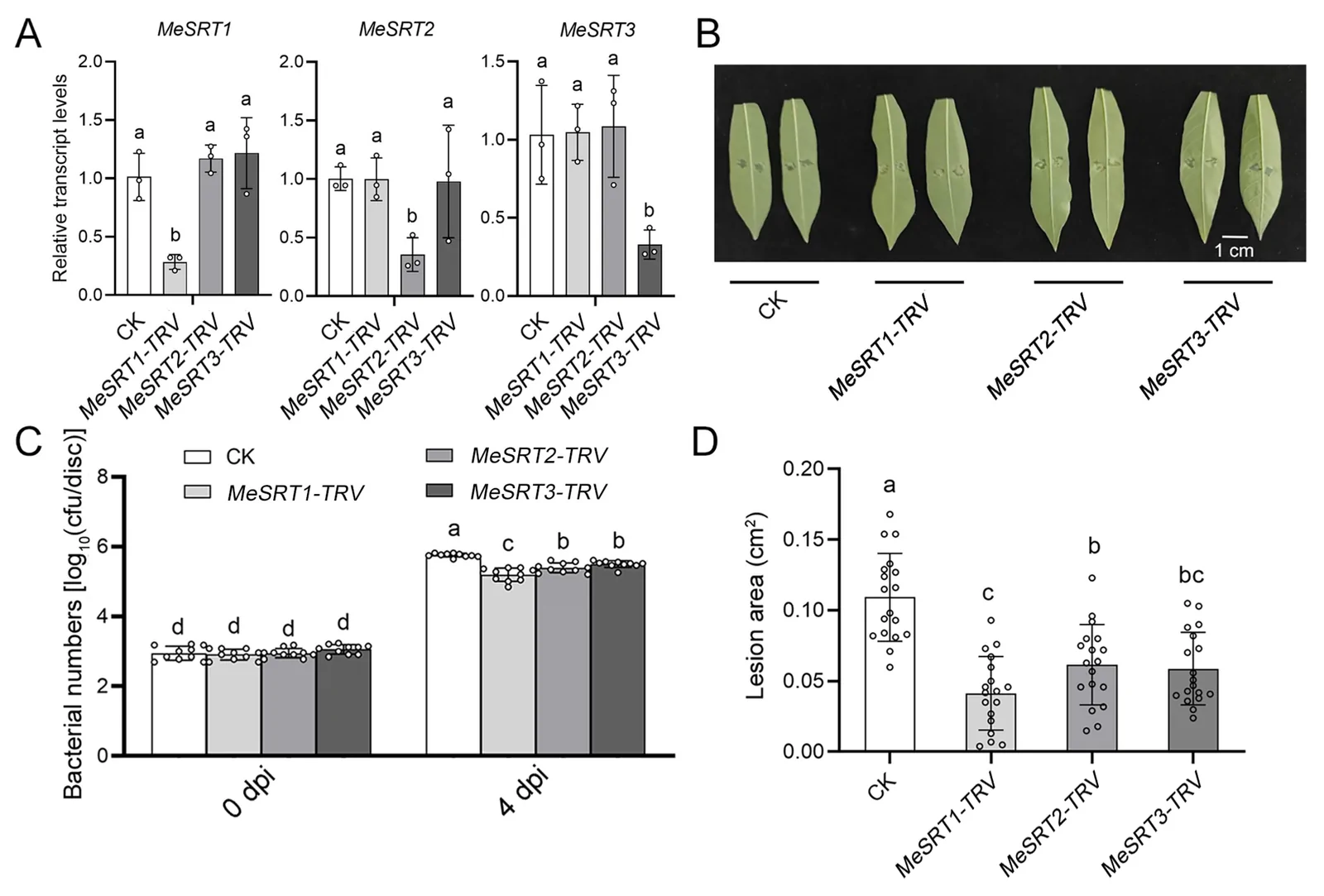
MeSRTs negatively regulate disease resistance in cassava. (**A**) The relative transcript levels of *MeSRTs* confirming gene silencing (*n* = 3). (**B**) The disease phenotypes of each *MeSRT-*silenced cassava. Scale = 1 cm. (**C**) The bacterial numbers in each *MeSRT-*silenced cassava (*n* = 10). (**D**) The lesion areas in each *MeSRT-*silenced cassava (*n* = 18). CK refers to empty silencing vector control. Results were presented as mean ± SD. Significant differences were calculated by Duncan’s range test by using SPSS (v21). Different letters represent significant differences (*p* < 0.05).

**Figure 3:**
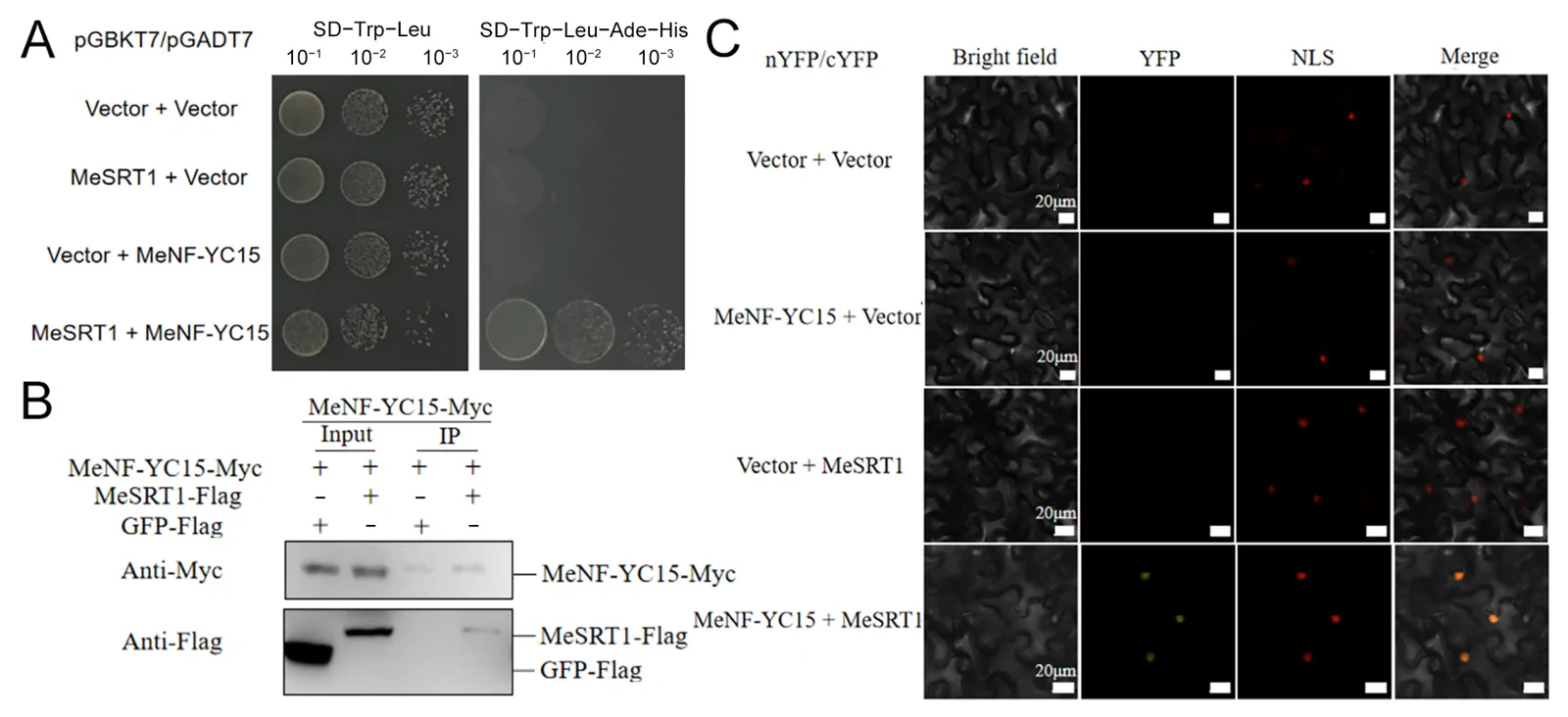
MeSRT1 interacts with MeNF-YC15. (**A**) Yeast two-hybrid assay indicates the interaction between MeSRT1 and MeNF-YC15. (**B**) Pull-down assay indicates the interaction between MeSRT1 and MeNF-YC15. (**C**) BiFC assay indicates the interaction between MeSRT1 and MeNF-YC15. Bar = 20 μm. Yellow color indicates the YFP signal. Red color indicates the NLS signal.

**Figure 4:**
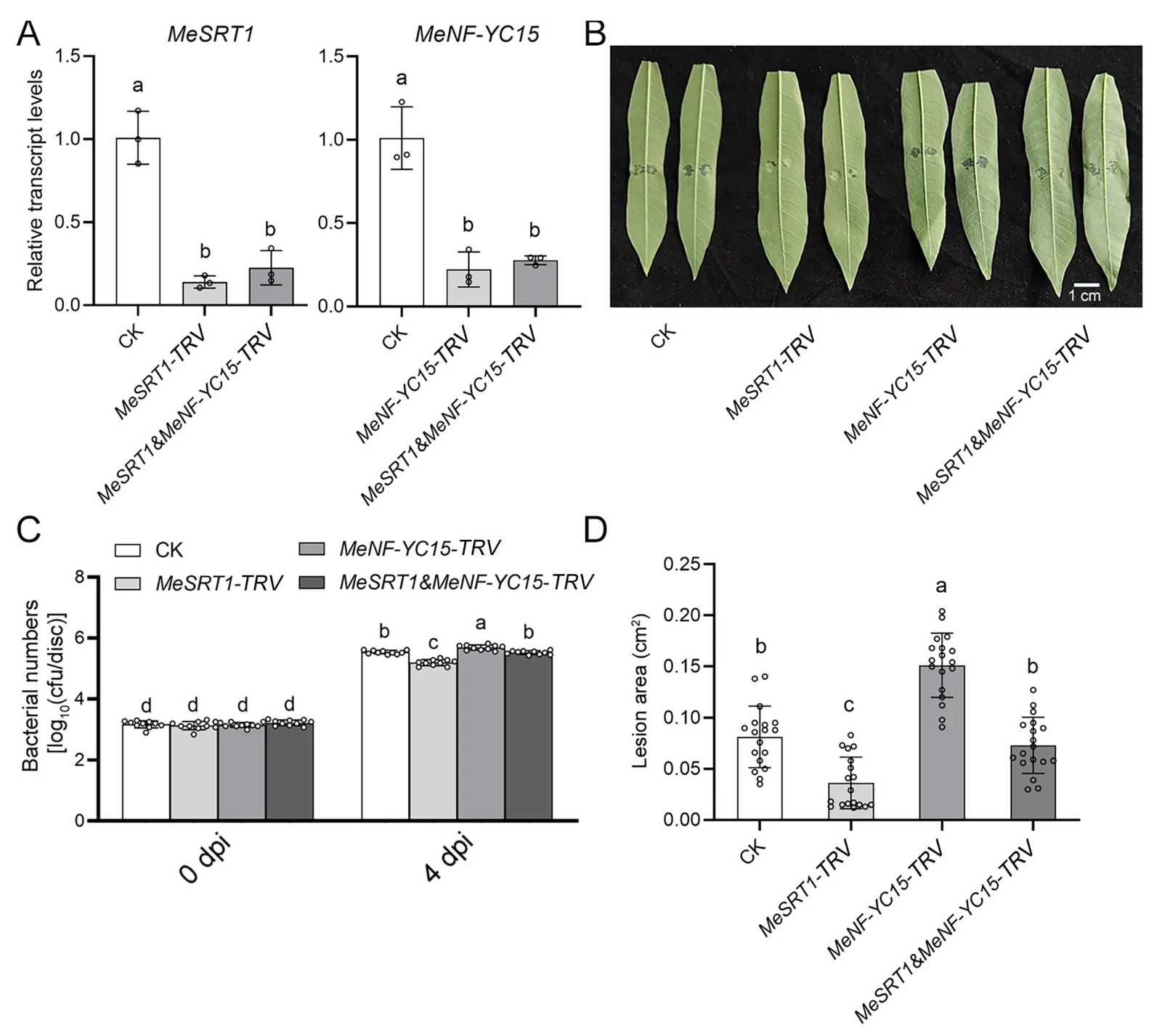
MeSRT1 and MeNF-YC15 antagonistically regulate disease resistance in cassava. (**A**) The relative transcript levels of *MeSRT1* or *MeNF-YC15* confirming gene silencing (*n* = 3). (**B**–**D**) The disease phenotypes (**B**), bacterial numbers (*n* = 10) (**C**), and lesion areas (*n* = 18) (**D**) of *Xpm* inoculated *MeSRT1-*silenced cassava, *MeNF-YC15-*silenced cassava, and *MeSRT1-MeNF-YC15-*co-silenced cassava. Scale = 1 cm. CK refers to the empty silencing vector control. Results are represented as mean ± SD. Significant differences were calculated by Duncan’s range test using SPSS (v21). Different letters represent significant differences (*p* < 0.05).

**Figure 5:**
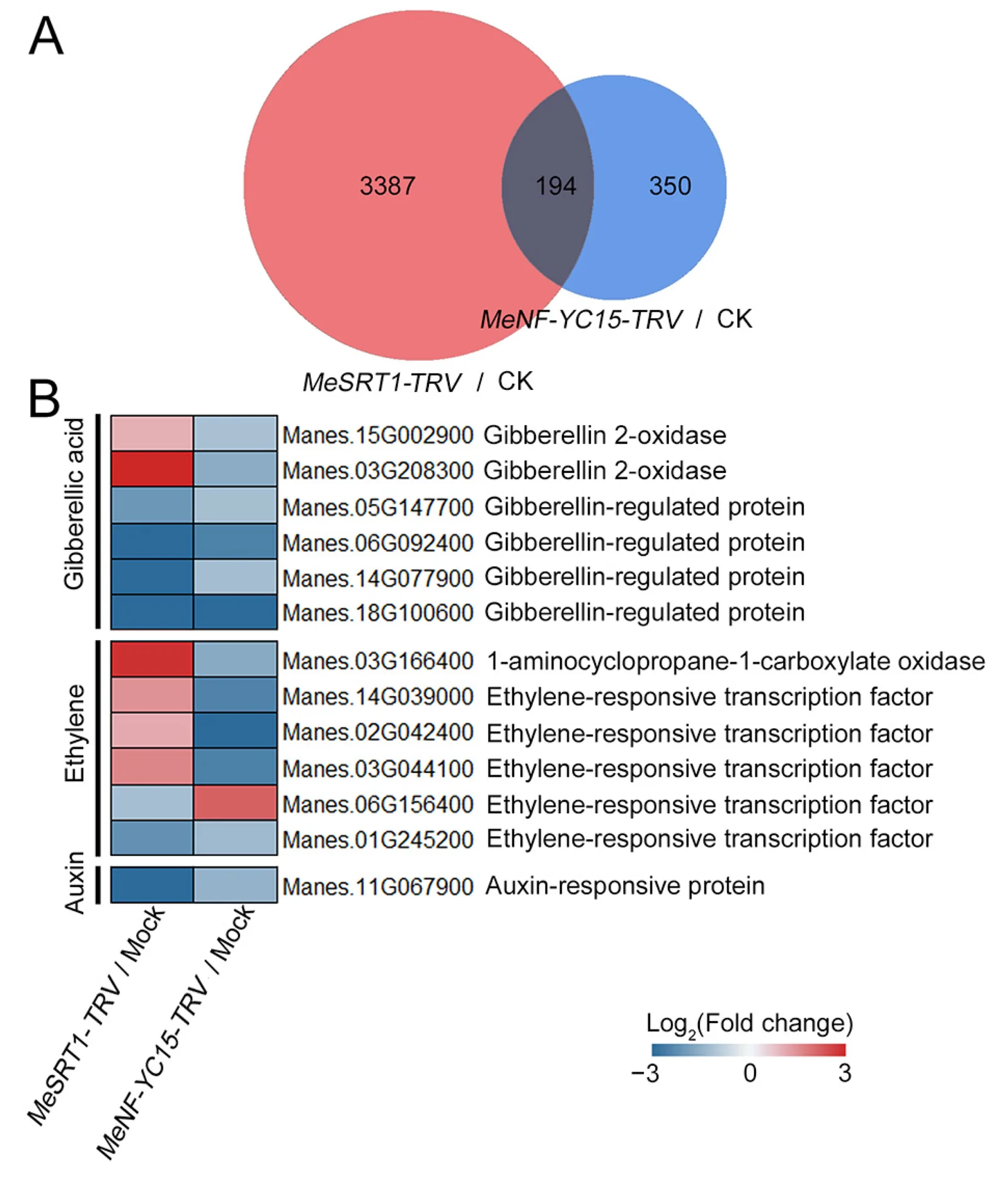
MeSRT1 and MeNF-YC15 commonly regulate the transcription of downstream genes in phytohormone signal transduction and metabolism. (**A**) Venn diagram showing the numbers of DEGs in *MeSRT1-*silenced cassava and *MeNF-YC15-*silenced cassava. CK represents the empty silencing vector control. 3387 DEGs were only identified in *MeSRT1-*silenced cassava. 194 DEGs were both identified in *MeSRT1-*silenced cassava and *MeNF-YC15-*silenced cassava. 350 DEGs were only identified in *MeNF-YC15-*silenced cassava. (**B**) Heatmap showing DEGs in phytohormone signal transduction and metabolism. DEGs were identified using |log_2_(Fold change)| ≥ 1 and adjusted *p* value < 0.05. The color scale indicates the value of log_2_(Fold change).

**Figure 6:**
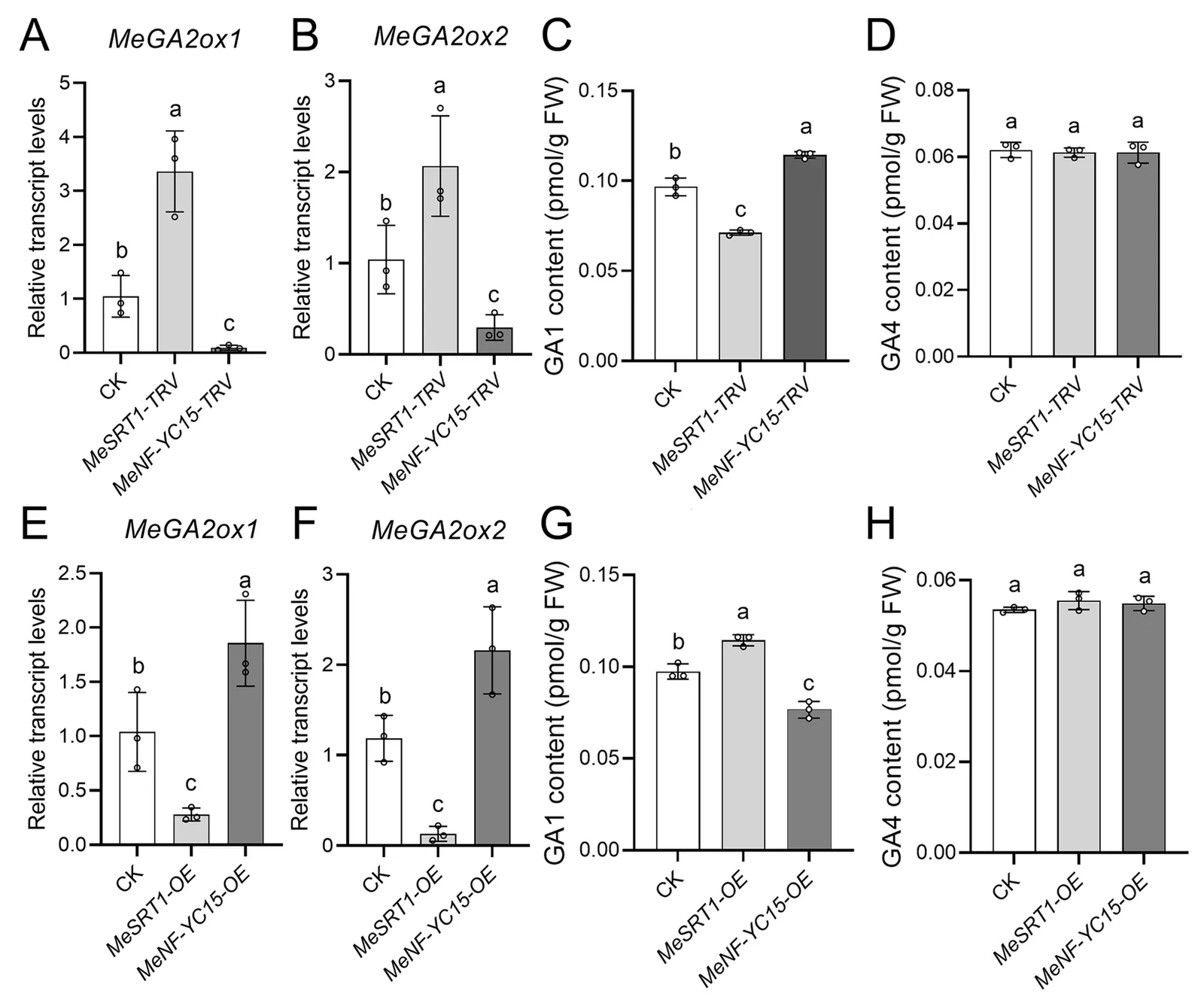
MeSRT1 and MeNF-YC15 antagonistically regulate GA1 content by regulating the transcription of *MeGA2ox1* and *MeGA2ox2*. (**A**,**B**) The relative transcript levels of *MeGA2ox1* (**A**) and *MeGA2ox2* (**B**) in CK and *MeSRT1-*silenced cassava. (**C**,**D**) The GA1 content (**C**) and GA4 content (**D**) in CK and *MeSRT1-*silenced cassava. CK (**A**–**D**) represents empty silencing vector control. (**E**,**F**) The relative transcript levels of *MeGA2ox1* (**E**) and *MeGA2ox2* (**F**) in CK and *MeNF-YC15* transiently overexpressing cassava leaves. (**G**,**H**) The GA1 contents (**G**) and GA4 content (**H**) in CK and *MeNF-YC15* transiently overexpressing cassava leaves. CK (**E**–**H**) represents the empty overexpression vector control. Results are represented as mean ± SD (*n* = 3). Significant differences were calculated by Duncan’s range test using SPSS (v21). Different letters represent significant differences (*p* < 0.05).

**Figure 7:**
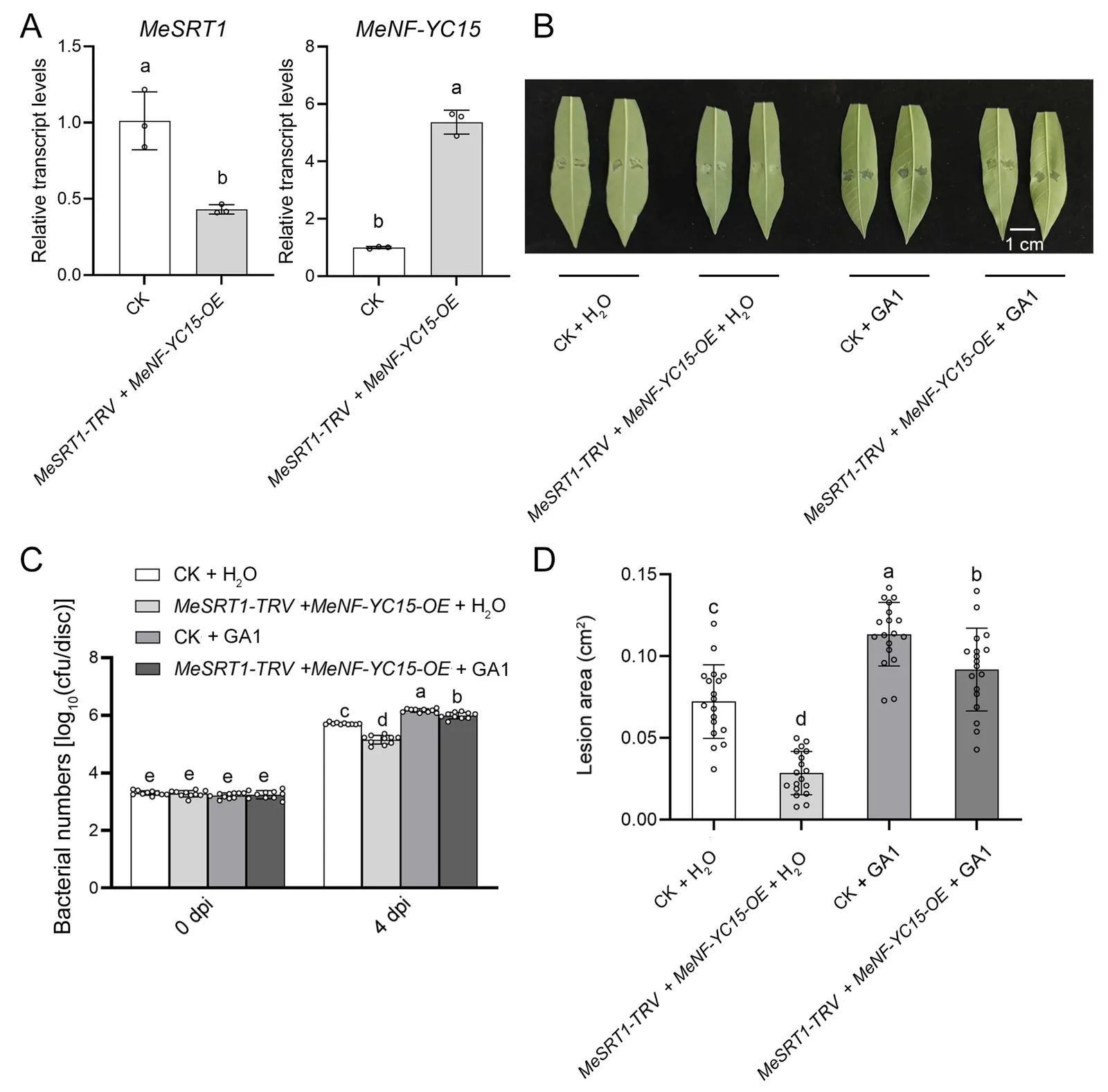
MeSRT1 and MeNF-YC15 commonly regulate cassava disease resistance through GA1 metabolism. (**A**) Relative transcript levels of *MeSRT1* or *MeNF-YC15* confirming gene silencing or overexpressing (*n* = 3). (**B**–**D**) Disease phenotypes (**B**), bacterial numbers (*n* = 10) (**C**), and lesion areas (*n* = 18) (**D**) of *Xpm-*inoculated cassava plants. Scale = 1 cm. CK represents the empty silencing vector and empty overexpressing vector control. Results are represented as mean ± SD. Significant differences were calculated by Duncan’s range test using SPSS (v21). Different letters represent significant differences (*p* < 0.05).

**Figure 8:**
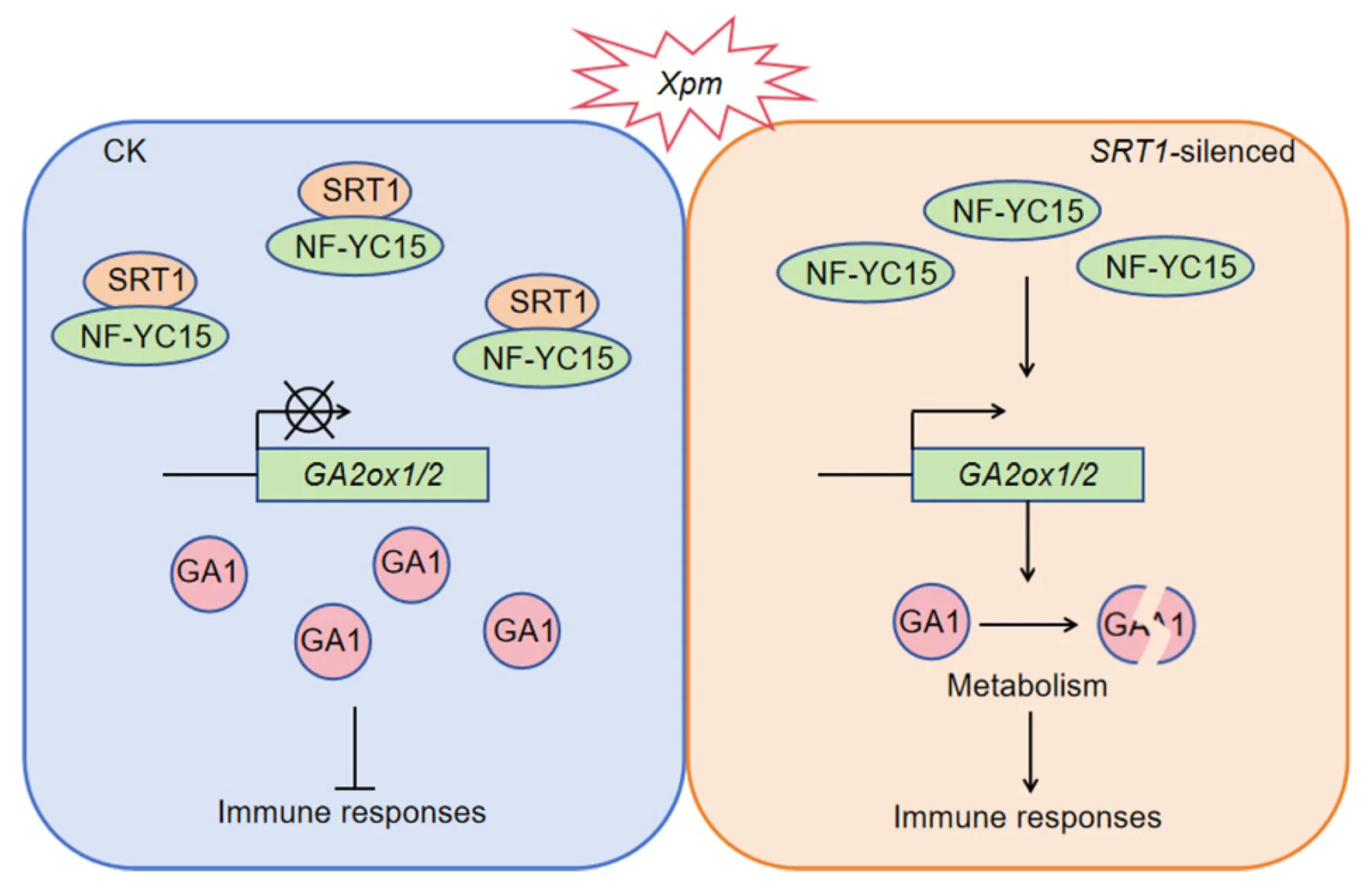
Schematic model of this study. In response to *Xpm* infection, MeSRT1 physically interacts with MeNF-YC15, which inhibits the transcription of *MeGA2ox1* and *MeGA2ox2*. This transcriptional inhibition prevents GA1 degradation and decreases immune responses in cassava. When *MeSRT1* is silenced, the transcription of *MeGA2ox1* and *MeGA2ox2* is activated, causing GA1 degradation, leading to disease resistance in cassava.

## Data Availability

The original contributions presented in this study are included in the article/Supplementary Materials. Further inquiries can be directed to the corresponding author.

## Supplementary Materials

The following supporting information can be downloaded at: https://www.mdpi.com/article/10.3390/plants15192898/s1, Figure S1. GA content in *MeSRT1-TRV* and *MeNF-YC15-OE* samples. The significant differences were calculated by Duncan’s range test using SPSS (v21). Different letters represent significant differences. Table S1. Information on MeSRT proteins in cassava. Table S2. Primers used for RT-qPCR. Table S3. Primers used for vector construction.
