# Supplementary material for: MeSRT1 Interacts with MeNF-YC15 and Negatively Mediates Resistance to Cassava Bacterial Blight via Regulation of Gibberellin Metabolism in Manihot esculenta Crantz
Source: Plants (Basel). 2026 Sep 22;15(19):2898. doi: 10.3390/plants15192898 (PMC13667163; doi:10.3390/plants15192898)
Supplement: Supplementary file 1 [file plants-15-02898-s001.zip › Figure S1.pdf]

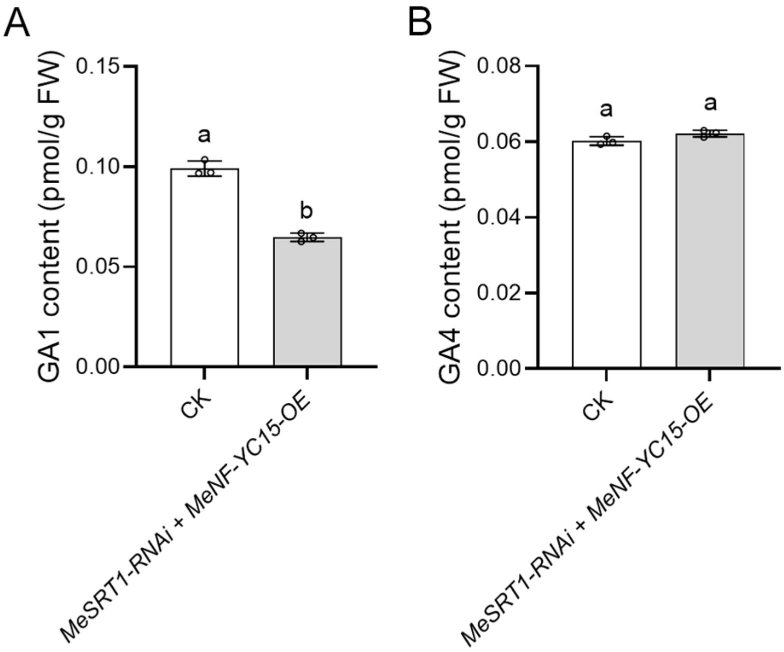

Figure S1. GAs content in *MeSRT1-TRV* & *MeNF-YC15-OE* samples. **(A-B)** GA1 contents (A) and GA4 contents. **(B)** in *MeSRT1-TRV* & *MeNF-YC15-OE* samples. CK represented empty silencing vector and empty overexpressing vector control. Results were shown as mean ± SD (n = 3). The significant differences were calculated by Duncan's range test by using SPSS (v21). Different letters represent significant differences ($p < 0.05$).
